# Drug development in Alzheimer’s disease: the path to 2025

**DOI:** 10.1186/s13195-016-0207-9

**Published:** 2016-09-20

**Authors:** Jeffrey Cummings, Paul S. Aisen, Bruno DuBois, Lutz Frölich, Clifford R. Jack, Roy W. Jones, John C. Morris, Joel Raskin, Sherie A. Dowsett, Philip Scheltens

**Affiliations:** 1 Cleveland Clinic Lou Ruvo Center for Brain Health, Las Vegas, NV USA; 2 University of Southern California, San Diego, CA USA; 3 Institute for Memory and Alzheimer’s Disease (IM2A) and ICM, Salpêtrière University Hospital, Paris University, Paris, France; 4 Department of Geriatric Psychiatry, Central Institute of Mental Health, Medical Faculty Mannheim, University of Heidelberg, Mannheim, Germany; 5 Department of Radiology, Mayo Clinic, Rochester, MN USA; 6 The Research Institute for the Care of Older People (RICE), Royal United Hospital, Bath, UK; 7 Knight Alzheimer Disease Research Center, Washington University School of Medicine, St Louis, MO USA; 8 Eli Lilly and Company, Indianapolis, IN USA; 9 Eli Lilly and Company, Toronto, Canada; 10 Department of Neurology & Alzheimer Center, VU University Medical Center, Amsterdam, Netherlands

**Keywords:** Alzheimer’s disease, Disease-modifying therapy, 2025

## Abstract

The global impact of Alzheimer’s disease (AD) continues to increase, and focused efforts are needed to address this immense public health challenge. National leaders have set a goal to prevent or effectively treat AD by 2025. In this paper, we discuss the path to 2025, and what is feasible in this time frame given the realities and challenges of AD drug development, with a focus on disease-modifying therapies (DMTs). Under the current conditions, only drugs currently in late Phase 1 or later will have a chance of being approved by 2025. If pipeline attrition rates remain high, only a few compounds at best will meet this time frame. There is an opportunity to reduce the time and risk of AD drug development through an improvement in trial design; better trial infrastructure; disease registries of well-characterized participant cohorts to help with more rapid enrollment of appropriate study populations; validated biomarkers to better detect disease, determine risk and monitor disease progression as well as predict disease response; more sensitive clinical assessment tools; and faster regulatory review. To implement change requires efforts to build awareness, educate and foster engagement; increase funding for both basic and clinical research; reduce fragmented environments and systems; increase learning from successes and failures; promote data standardization and increase wider data sharing; understand AD at the basic biology level; and rapidly translate new knowledge into clinical development. Improved mechanistic understanding of disease onset and progression is central to more efficient AD drug development and will lead to improved therapeutic approaches and targets. The opportunity for more than a few new therapies by 2025 is small. Accelerating research and clinical development efforts and bringing DMTs to market sooner would have a significant impact on the future societal burden of AD. As these steps are put in place and plans come to fruition, e.g., approval of a DMT, it can be predicted that momentum will build, the process will be self-sustaining, and the path to 2025, and beyond, becomes clearer.

## Background

Increasing life expectancy has produced a dramatic rise in the prevalence, and thus impact, of aging-associated diseases including dementia. Alzheimer’s disease (AD) is by far the most common dementia in late life. It is currently estimated that 46.8 million people worldwide have dementia with an estimated global cost of dementia care at US$818 billion in 2010 [1]. By 2030 it is estimated that there will be 74.7 million people with dementia, and the cost of caring for these individuals could rise to some US$2 trillion. In the absence of effective therapies, the estimated number of people with dementia will reach 131.5 million by 2050.

Global leaders have set a deadline of 2025 for finding an effective way to treat or prevent AD [2]. In the United States in late 2010/early 2011, the National Alzheimer’s Project Act (NAPA) was passed and signed into law [3]. It required the creation of a national strategic plan to address the rapidly escalating AD crisis and the coordination of AD efforts across the federal government. The overarching research goal of the project is to “prevent or effectively treat Alzheimer’s disease by 2025”. In December 2014, the G8 stated that dementia should be made a global priority with the aim of a cure or approved disease-modifying therapy (DMT) available by 2025. Although the politically initiated 2025 deadline may not have been based on scientific principles of disease research or the realities of drug development, it has become a rallying cry for researchers and advocates as they endeavor to find innovative ways to develop drugs to successfully achieve the 2025 goal.

Despite the evaluation of numerous potential treatments in clinical trials [4, 5], only four cholinesterase inhibitors and memantine have shown sufficient safety and efficacy to allow marketing approval at an international level. These five agents are symptomatic treatments, temporarily ameliorating memory and thinking problems, and their clinical effect is modest; they do not treat the underlying cause of AD and do not slow the rate of decline [6].

Over the past decade, the focus of drug discovery and development efforts has shifted toward DMTs for AD; that is, treatments whose aim is to affect the underlying disease process by impacting one or more of the many brain changes characteristic of AD. These treatments could slow the progression of the disease or delay its onset. Less encouraging is that, over the same time period, numerous candidate agents have failed in clinical development, and no DMTs have shown a drug-placebo difference in Phase 3 studies or received marketing approval [7].

While AD drug failures to date are likely, in part, because the drugs tested lacked sufficient target engagement or had toxic effects [8], efforts to bring new AD drugs to market have been hindered by a number of challenges—incomplete understanding of AD pathogenesis, the multifactorial etiology and complex pathophysiology of the disease, the slowly progressive nature of AD, and the high level of comorbidity occurring in the elderly population [9]. Further challenges exist in the clinical trial environment because overt clinical symptoms are not evident until considerable change has occurred within the brain, the most appropriate outcome measures have not been widely agreed upon, there is difficulty establishing and coordinating global clinical trial networks, and strategies for identifying and recruiting trial participants are time- and cost-intensive [10]. As we move to treating earlier in the disease continuum, there is more sensitivity around risk associated with drug use; drug failures may be the result of studying too low a dose in an effort to decrease the occurrence of side effects, including amyloid-related imaging abnormalities (ARIA), which are still poorly understood.

In this paper, we discuss the path to 2025, and what is feasible in this time frame given the realities and challenges of AD drug development. We focus on development of DMTs for individuals with early disease, which may be more amenable to disease modification and most likely to fulfill the 2025 mandate of meaningful new therapy. Early disease is defined here as mild cognitive impairment (MCI) due to AD (National Institute on Aging-Alzheimer’s Association (NIA-AA) criteria [11]) or prodromal AD (International Working Group (IWG) criteria [12]). Clinical study of individuals with preclinical AD (asymptomatic persons at increased risk for symptomatic AD) are being pursued but their longer time frames and measurement and regulatory uncertainty makes them less likely to contribute to reaching the 2025 goal; this topic is not discussed in depth here, but it is recognized that the challenges are likely to be similar, though on a greater scale, to those associated with development of treatments for MCI. Identifying DMTs for use in preclinical populations will take longer given uncertainties about disease progression, clinical outcomes, biomarkers, and regulatory views.

We provide examples of current activities and practices to help address these complex challenges and briefly discuss activities that need to start now but may not have direct impact until after 2025. We intend to set the stage for continuing progress in the AD drug development field, and to stimulate further discussion and action for impact in the short term to fulfill the 2025 goal and long term for continuing development in the AD space beyond 2025.

## AD drug development—current status

For a DMT, after preclinical development and initial characterization of an AD agent, Phase 1 takes approximately 13 months, Phase 2 approximately 28 months, and Phase 3 approximately 51 months, followed by regulatory review of approximately 18 months [13]. Including preclinical development, the total development time reaches 160 months (more than 9 years). The cost of developing a DMT for AD, including the cost of failures, is estimated at $5.7 billion in the current environment [13].

**Fig. 1:**
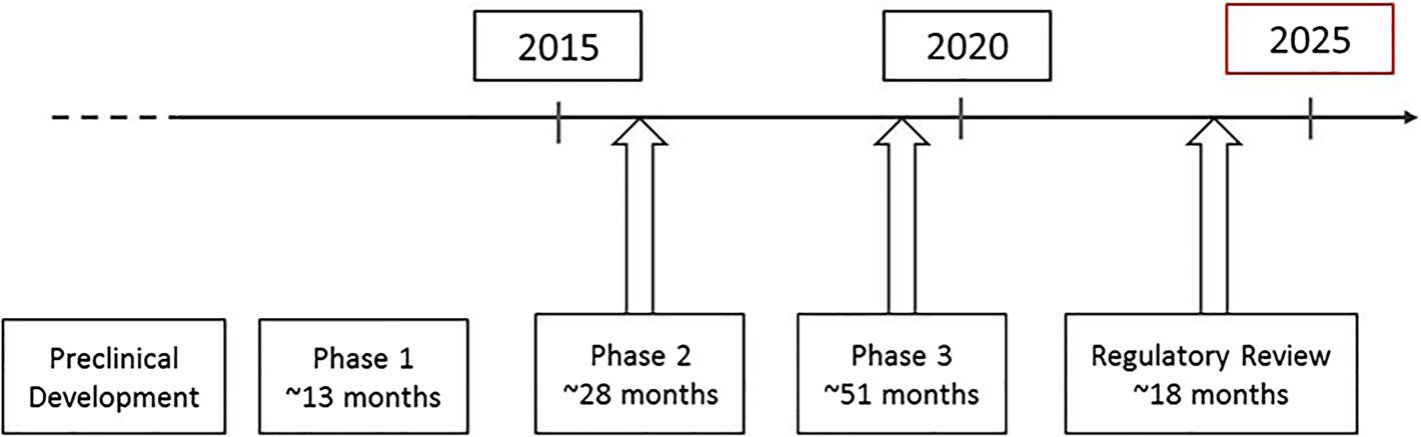
Current timeline for clinical development of disease-modifying drugs for approval by 2025 [8]

To determine if availability of DMTs by 2025 is a realistic goal, we can work backwards from 2025 (Fig. 1). If the current timeline remains unchanged, approval of a DMT by 2025 requires that the agent be under regulatory review by 2023/2024. Thus, Phase 3 studies will need to start by 2019 to allow sufficient time for recruitment, treatment in trial and analysis/interpretation, and Phase 2 will need to start in 2016/2017. Therefore, for approval by 2025, potential AD DMTs need to be in late Phase 1 now, and most compounds with a chance of success by 2025 will currently be in Phase 2 or 3.

The number of DMTs in clinical trials is small considering the need. Currently in Phase 1 through Phase 3 development, there are approximately five active immunotherapies, 11 passive immunotherapies, and 55 small molecules (including but not limited to DMTs) [14]. The main targets are amyloid, followed by inflammatory mediators/factors and tau, and the dominant approach is passive immunization targeting amyloid. Reviews of select DMTs in development are provided by Scheltens et al. [15] and Hendrix et al. [16]. Attrition is high, and few compounds, at best, will be approved in the 2025 time frame.

## Accelerating AD drug development to 2025: overcoming key challenges

Hastening new drug development with a view to implementing drug treatment and prevention strategies by 2025 will require advances within the clinical trial setting as well as progress in the regulatory environment. We discuss specific measures that are needed in these areas, and provide examples of where they are already being implemented.

### Clinical trial environment

Speeding up clinical development, particularly in Phase 2 and 3, requires innovations and improvements in study design and trial execution, as well as more effective recruitment measures and disease detection/monitoring.

#### Trial design and operation

Traditionally, individual study phases are completed before moving to the next phase of the study. However, as has been the case in immunotherapy development, combined Phase 1/2 clinical trials may speed development; that is, instead of conducting a Phase 1 trial for toxicity and a separate Phase 2 trial for efficacy, it may be appropriate to integrate these two phases into one study of individuals with AD. Study sponsors can consider an adaptive Phase 2/3 study design, whereby accumulating trial data are used to guide modification of one or more specified aspects of the study design, for example reducing the number of dose arms, or extending or shortening the length of the trial without undermining its validity and integrity. Use of such an adaptive trial design places greater emphasis on Phase 2 learnings as guides to pharmaceutical decision-making (for example, whether to continue development of an investigational drug). While AD drug development could be reduced by months or even years using an adaptive design, there is some skepticism about its value with concern of erroneous trial modifications as a result of the “noise” with our current cognitive measures as well as with non-validated biomarkers. Intensive study of novel study designs will be required to understand their appropriate role within the AD trial setting and potential for drug development acceleration.

While the advance of a drug from Phase 2 to Phase 3 is a step closer to the goal, there is a risk of wasting both time and money if this decision is based on secondary analysis and subgroup findings when the primary endpoint is not met in Phase 2. Rigorous adherence to pre-specified outcomes and avoidance of over-interpreting subgroup data, as well as greater understanding of the test agent in Phase 2 and appropriate primary endpoint selection, are crucial and will help preserve resources for agents with a higher likelihood of success.

Patient recruitment and enrollment are the most time-consuming aspects of the clinical trial process and this is particularly so for Phase 3 studies where sample sizes for AD trials of DMTs are often more than 1000 participants. Recruitment to AD trials is notoriously slow, and can take years for AD Phase 3 trials. Speeding up recruitment is a key area where clinical development timelines could be impacted, reducing Phase 2 and Phase 3 study times by many months.

Efforts are needed to increase recruitment include expanding public and healthcare professional (HCP) understanding of AD as well as increasing their awareness of opportunities for AD trial participation, whether in currently recruiting trials or in future trials through voluntary inclusion on AD registries. HCPs also need to be more informed and prepared for screening of patients and referral to memory centers. Patient networks and advocacy groups will play an increasingly important role in engaging the general population and raising their awareness of AD and dementia, as well as AD trial participation opportunities, for example through the creation of more Dementia Friendly Communities (DFCs) (e.g., The Heart Ring Movement campaign in Japan, and The Dementia Friends program initiative launched by the Alzheimer’s Society in the UK). This will be particularly important in countries where there is currently a less enthusiastic attitude towards clinical trial participation. Voices of politicians, entertainment celebrities, sports figures, religious leaders, and other stakeholders may ultimately be necessary to fully galvanize populations into action.

To streamline enrollment in AD clinical trials and speed up recruitment, registries of healthy aged and symptomatic individuals are vital. As well as providing a repository for information about people with a specific condition, registries provide a connection between people who are willing to participate in research and those recruiting for studies. More advanced registries with standardized demographic, genetic, biologic, cognitive, and environmental information on potential participants could reduce the length of clinical trials further after the initial eligibility testing has been established. The availability of serial assessment information within a database could permit selection of trial participants based on disease trajectory. The availability of a trial-ready cohort in which both amyloid and cognitive status are known could potentially provide the greatest time saving in treatment development; however, the cost of establishing such cohorts is significant. Finding mechanisms to avoid “labeling” participants, ensuring security and privacy of data, and respecting transgenerational inferences are all key to development of successful registries.

AD registries currently in place to help with more effective recruitment to clinical trials include:

- The Alzheimer’s Prevention Registry [17]
- The Dominantly Inherited Alzheimer Network Trials Unit (DIAN-TU) Expanded Registry [18], to aid recruitment of individuals at risk of having a gene mutation that causes dominantly inherited AD to trials of potential DMTs;
- The Brain Health Registry [19], a global online registry for anyone age 18 years and older interested in research of new treatments for AD, and other conditions that affect brain function (includes opportunities for clinical trial participation);
- The Global Alzheimer’s Platform (GAP) initiative [20, 21];
- The Cleveland Clinic Healthy Brains Registry (healthybrains.org) [22];
- The Alzheimer's Disease Cooperative Study (ADCS) [23], a federal-university collaboration and also part of the Alzheimer Prevention Initiative; and
- The Join Dementia Research initiative [24] in the UK.

Linking individual registries will further increase the potential participant pool, and that is beginning to occur. The Brain Health Registry has recently partnered with GAP to dramatically increase the registry’s database size to tens of thousands of new registry members; under the joint effort, supporters hope to have 40,000 people registered before the end of 2016. 

Other channels of recruitment, including community screenings, advocacy forums, educational programs, social media campaigns, and use of conventional media (television, radio, newspapers), can function to refer potential participants to registries or directly to trial sites.

Trial execution can be far more efficient if there is an integrated standing network of clinical trial sites. These clinical trial platforms may include local disease registries, trial-ready cohorts, and optimized administrative procedures, and they are increasingly being pursued as a way to assure less redundancy (e.g., through acceptance of a standardized budget and contract language and annual rather than trial-specific rater training and certification) and greater speed compared to the existing clinical trial procedures. GAP, for example, hopes to reduce the clinical testing cycle by 2 years or more through the development of certified clinical trial sites and, as already discussed, registries with cohorts of trial-ready patients.

Clinical trial execution is dependent upon Institutional Review Board (IRB)/Ethics Committee (EC) approval, and with multi-center trials this invariably means approval by numerous individual IRBs/ECs. The burden on IRBs/ECs and on sponsors and clinical investigators who are seeking review for multicenter trials is considerable; there is unnecessary expense, duplication of effort, and delays. Greater reliance on a centralized (even national) IRB review process could reduce this time-consuming problem.

Fostering stronger ties between clinical practice and research could also speed trial recruitment and increase trial efficiency. Comprehensive AD Centers, whereby clinical activities and research efforts are brought together so that patient care and clinical study of AD can occur in a more integrated environment, are being established. Examples include: The Gérontopôle in Toulouse, France [25]; the Salpêtrière Dementia Research Center, in Paris, France; the Amsterdam Dementia Cohort [26]; the German Dementia Competence Network [27]; the Cleveland Clinic Lou Ruvo Center for Brain Health, USA; and the University of Southern California Alzheimer’s Therapeutic Research Institute (USC ATRI), USA.

#### Detection and monitoring of disease—clinical assessment tools

As we shift clinical focus from the study of symptomatic treatments in populations with more advanced disease to DMTs in populations with earlier disease, several challenges arise with the use of currently available assessment tools. While DMTs will potentially slow cognitive decline, they may not provide immediate improvement like existing symptomatic treatments. Cognitive instruments, such as the Alzheimer’s Disease Assessment Scale-Cognitive subscale (ADAS-Cog), Mini-Mental State Examination (MMSE), and neuropsychological test items show relatively little change over time in individuals with early symptoms of disease, primarily due to ceiling effects in many of the items that make up these scales (i.e., there are ceiling effects, and parts of the test do not properly capture subtle changes over time). In addition, since functional worsening occurs later in the disease process and seems to follow cognitive decline [28, 29], individuals with no or minimal cognitive symptoms are likely to have no functional compromise, limiting the relevance of functional and global assessments.

There is a need for more sensitive and responsive instruments for use in these early stages of AD; in particular, more sensitive and specific cognitive assessment tools to capture subtle clinical decline are required to identify individuals with minimal symptoms and discern treatment effects among participants with earlier disease. As a result of the early involvement of the medial temporal lobe in AD pathogenesis, a specific memory profile has been reported in AD that is characterized by a diminished free recall ability that is only marginally improved by cueing (amnestic syndrome of the hippocampal type) [30]. The Free and Cued Selective Recall Reminding Test (FCSRT) can be used to detect impairment of free and cued recall and identify patients with MCI with high sensitivity and specificity [31, 32]. Computerized neuropsychological assessment may offer a greater degree of sensitivity, and assessments can be performed more frequently so within-subject changes can be detected more easily. Unfortunately, computer experience can influence computerized test performance and this is likely to be more of a challenge in elderly individuals. Exploration of these emerging alternatives may lead to new standard assessments in the AD trial environment.

Functional assessment remains a key challenge in AD drug development, and more sensitive tools to assess deficits in function are urgently needed. Function is commonly assessed using Activities of Daily Living (ADL) measurement instruments. ADL is divided into Basic Activities of Daily Living (BADL), which includes self-maintenance skills such as bathing, dressing or eating, and Instrumental Activities of Daily Living (IADL), which involves more complex activities such as using public transportation, managing finances, or shopping. These instrumental activities generally require a greater complexity of neuropsychological organization and are therefore likely to be vulnerable to the early effects of cognitive decline. Although there is incomplete agreement about which IADL domains are typically impaired in prodromal AD and which types of instruments may detect those best, it is clear that activities requiring higher cognitive processes are the most consistently affected items. New instruments for assessment of IADL functioning including items measuring the domains of financial capacities, keeping appointments, task completion time, decision making, speed of performance, and task accuracy [33] are needed.

Development and validation of new scales de novo is a long process. Recent efforts have focused on developing composites, which capture only those components from existing scales that have the ability to discern decline in early AD populations; for example, by eliminating items from the ADAS-Cog that appear less sensitive to early changes and combining the remainder with items from other instruments of cognition and/or function, sensitivity to change and reduced variability can be achieved. The Food and Drug Administration (FDA) has also indicated that a single composite outcome may be appropriate for trials of individuals with MCI/prodromal AD [34]. Composites for the study of individuals with MCI include the integrated Alzheimer’s Disease Rating Scale (iADRS) [35], comprised of the scores from two widely accepted measures, the ADAS-Cog and the ADCS-instrumental Activities of Daily Living (ADCS-iADL), and the AD Composite Score (ADCOMS) [36], comprised of four ADAS-Cog items, two MMSE items, and all six Clinical Dementia Rating-Sum of Boxes (CDR-SB) items. ADCOMS which has been demonstrated to have improved sensitivity to clinical decline over individual scales in individuals with earlier symptoms of AD. New tools are also needed for preclinical AD trials. The ADCS-Preclinical Alzheimer Cognitive Composite (ADCS-PACC) is a cognitive composite and the primary outcome measure in the Anti-Amyloid Treatment in Asymptomatic AD (A4) Trial of individuals with pr eclinical AD [37]. Other instruments for preclinical trials are being assessed.

Ultimately, the goal is to ensure that any assessment tool employed provides clinically meaningful information. In studies of populations with early AD symptoms, it might be appropriate to consider cognition alone as a primary endpoint, and this may require a better understanding of the clinical meaningfulness of cognitive changes and their ability to predict functional decline. A composite composed of appropriate cognitive and functional components would also be of use. More sensitive tools, whether cognitive or functional, could help speed clinical development by shortening recruitment time and reducing the required sample size.

#### Detection and monitoring of disease—biomarkers

Biomarkers have become instrumental in efficient clinical development of drug entities for many diseases, assisting with appropriate patient selection, testing target engagement by a drug, and monitoring disease progression. In the AD field, biomarkers will be essential to speed clinical development.

Diagnostic AD markers are considered those reflective of AD pathology. In this realm, the focus thus far has been on cerebrospinal fluid (CSF) markers--- Aβ_42_ and tau (total tau or phosphorylated tau)--- and positron emission tomography (PET) imaging with amyloid or tau tracers to provide information on the extent of amyloid plaque burden or tau neurofibrillary tangles in the brain [12, 38]. Use of diagnostic markers is essential to ensure enrollment of individuals who have AD pathology; clinical diagnosis alone of AD dementia is not always accurate. Approximately 25 % of subjects diagnosed clinically with mild AD dementia have been shown to be amyloid-negative [39] and the proportion is even higher in MCI [40]. Diagnostic biomarkers, however, need to be inexpensive and simpler to use if they are to be widely integrated. Topographical biomarkers are used to identify downstream brain changes indicative of AD pathology (brain regional structural and metabolic changes) [12]. They include magnetic resonance imaging (MRI)-related biomarkers, for example structural MRI to assess hippocampal atrophy, ventricular volume, whole brain volume, and cortical thickness. While useful as disease progression markers in trials, they lack the specificity of diagnostic markers and may not be helpful in early stages of disease.

In light of the challenges with clinical endpoints and the protracted and unpredictable clinical course of AD, it is crucial to have access to surrogate biomarkers that could provide an early indication that a drug is having an effect that will ultimately lead to cognitive and functional improvements; no qualified surrogates for AD trials are currently available. The use of surrogate markers would make clinical trials of potential DMTs more efficient. Their use would enable better decisions about which compounds (from a number of closely related candidates) to advance and at what dosage, thus reducing the overall risk of failure. The more clinical trials that incorporate potential surrogates, the sooner the discovery and qualification of a surrogate marker can be expected. Once it is known that a surrogate endpoint predicts clinical benefit, the surrogate endpoint may be used to support additional approvals. Establishment of surrogate status for biomarkers takes years; as a result, they are unlikely to be available to assist reaching the 2025 goal.

A target engagement biomarker helps determine whether the study drug has engaged its target in the disease process and thus has the opportunity to produce a clinical benefit. They are most useful for eliminating compounds that have inadequate engagement to effect a clinical change, thus freeing resources to invest in more promising agents. Their use in Phase 2 would help eliminate ineffective drugs so that failures in Phase 3 are reduced.

### Repurposed drugs

Repurposed agents are drugs that have been approved for another indication but may have pharmacological effects relevant to the treatment of AD [41]. Repurposed agents with possible effects in AD include, but are not limited to, statins, anti-hypertensives, cancer treatment agents, and anticonvulsants [42–44]. Repurposed agents have the potential of accelerating the AD drug development timeline. They have already been through preclinical toxicology assessments; Phase 1 human safety, tolerability and pharmacokinetic assessments; Phase 2 safety and efficacy studies for the original indication; Phase 3 studies for the original indication, and regulatory review for the original indication. Development of a repurposed agent for use in the AD field could begin with a Phase 2 proof-of-concept and dosing study for AD, thus avoiding the time and expense of preclinical development and Phase 1. Challenges do exist, however. While safety and tolerability of these drugs are well known, they will not have been used in AD populations where vulnerabilities may differ. In addition, many of these agents have no or limited patent protection and intellectual property challenges may decrease the interest of pharma in investing in their development [45]. This alternate pathway for AD drug development has promise but is not likely to have a major impact on the 2025 AD treatment goal.

### Regulatory environment

With a shift in the focus of AD drug development to earlier stages of disease, both the FDA and the European Medicines Agency (EMA) are putting increasing emphasis on opportunities in this realm and have issued draft guidances addressing drug development for AD [34, 46]. We provide both AD-specific and more general examples of where regulatory changes have, or could help, speed AD drug development.

#### Accelerating the review process

Accelerating the review process could shorten the overall development cycle by several months. At the FDA, the update of the Prescription Drug User Fee Act (PDUFA) V in 2013 has resulted in shorter review times with review time goals met more often than in previous years, and average review time reduced; in 2012, the median review time was 10 months, versus 8.5 months in 2015 [47]. The FDA has introduced expedited programs for treatments that address unmet medical needs in serious diseases [48]:

- Accelerated Approval is based on a surrogate endpoint that is “reasonably likely to predict clinical benefit” with clinical benefit verified through post-approval (Phase 4) testing; it has been used to approve over 90 new drugs and biologics, for example, in AIDS and cancer;
- Priority Review provides a shortened FDA review goal (6 vs 10 months, after 2 months filing) and is based on study findings;
- Fast Track Designation involves the FDA working closely with drug sponsors to facilitate submission of acceptable drug development plans; once the sponsor begins to develop its marketing application data, it can submit the data to the FDA for “rolling review,” rather than the usual process of submitting the entire marketing application at once. This could potentially save 1 to 2 months if the FDA begins reviewing initial portions of a rolling submission when they are available. While fast track does not guarantee a shorter review process, fast track submissions show more promise for receiving priority review, pending study findings. Of the DMTs currently in development, a few (less than 10) have received fast track designation;
- Breakthrough Therapy Designation whereby the FDA provides intensive guidance on an efficient drug development program, beginning as early as Phase 1. To date, more than 100 drug development programs, predominantly in oncology, have been granted breakthrough therapy designation. In cancer drug development, breakthrough therapy designation was associated with a 2.2-year reduction in time to approval [49]; in that study, all drugs receiving breakthrough designation received priority review and most received fast track designation. Going forward, this path could be applicable to AD drug programs.

In July 2015, the EMA revised their guidelines [50] on the implementation of accelerated assessment and conditional marketing authorization to accelerate access to medicines that address unmet medical needs. The revisions include more detailed guidance on how to justify fulfilment of major public health interest, allows for a faster assessment of eligible medicines by EMA scientific committees, and emphasizes the importance of early dialogue with the EMA so that accelerated assessment can be planned well ahead of the submission. The guidelines also specify information on conditional marketing authorization which allows for the early approval of a medicine on the basis of less complete clinical data than normally required if the medicine addresses an unmet medical need and targets a serious disease.

#### Study endpoint considerations

As noted above, efficient AD clinical development requires that study endpoints are most appropriate for the mechanism of action of the drug being tested (e.g., disease modifying or symptomatic treatment) and the stage of disease being targeted (e.g., preclinical, MCI, AD dementia). In the regulatory setting, it may be appropriate to consider drug approval based on a cognitive outcome only rather than cognition and function in these earlier stages of disease. The difficulty in showing a drug effect on functional endpoints in those with earlier disease is recognized by the FDA [34] and EMA [46]. It may be feasible to use a cognitive primary endpoint as an intermediate or surrogate endpoint for an accelerated approval, followed by a continuation of the study or a separate study demonstrating persistence of benefit to support a later approval with standard endpoints.

Eventually, AD drug development time could be shortened through acceptance of more sensitive clinical endpoints and biomarkers, particularly surrogate markers for efficacy, as data become available to support their predictive utility for clinical benefit.

#### Number of pivotal studies

Approval pathways that allow two pivotal studies to use two different populations in the AD continuum (e.g., one study in mild AD and one study in prodromal AD rather than two trials for each) are being considered by regulators [46]. This could allow availability to a broader patient population at initial approval, thus shortening the time to availability for one of the populations by 3 to 5 years. Taking this a step further, if conditional approval was based on findings from a single trial in one AD population with the requirement to test the drug more broadly after approval, Phase 3 development could be shortened considerably, though with a potential risk of use of a drug that is ineffective in some populations.

### Other considerations in accelerating AD drug development

While we have focused on immediate efforts to hasten clinical development, simultaneously there needs to be advancement in other areas that, while not necessarily having direct impact on the 2025 goal, will have long-term consequences in AD drug development.

#### Basic research

There are currently less than 25 agents in Phase 1 AD development [14, 51]. In light of the high attrition over the course of drug development, this is not adequate to ensure that new, effective, and diversified therapies successfully complete Phase 3 development. With the time frames required for AD drug development (Fig. 1), new research investments in the basic science of AD will not have their impact until after 2025. Continuing basic research efforts are fundamental to eventual clinical advancement. We will need to focus efforts, including funding, on furthering understanding of disease pathogenesis to identify treatment targets, effectively determine risk, measure disease activity, and treat and ultimately prevent disease. This is not without its challenges, particularly since the underlying neuropathology of AD precedes symptom onset by 15 to 20 years. New investment in federally funded basic science and industry-based research will be critically important to advancing the AD field. Also important is the diversification of models in which efficacy can be assessed. AD drug efficacy is typically explored using transgenic mice but they recapitulate only a limited part of the AD biology (e.g., amyloidosis), and efficacy has not been predictive of cognitive benefit in the human setting. Improved reproducibility of animal model observations, greater focus on animal systems as models for only a portion of the AD process, and increased emphasis on how best to translate animal observations to human trials are all areas that can increase the value of animal models in AD drug development. Newer models include human-derived induced pluripotent stem (iPS) cells, which can increase predictive confidence of the observations in nonclinical stages of development [52, 53].

Together with basic research in the AD field, we need to ensure that any new knowledge is rapidly integrated into clinical development. The scientific knowledge available when compounds currently in Phase 3 were in early development is now nearly a decade old. Shortening the development cycle times will lessen the gap between scientific discovery and clinical development, thus allowing more scientifically informed drug discovery and development.

#### Drug pipeline and combination therapy

In concert with basic research efforts, we need greater diversity in the preclinical pipeline, with development of novel therapeutic approaches and targets. More diversity will increase the likelihood that success in preclinical development will translate to success in clinical development (i.e., higher probability that at least one candidate will succeed), thereby reducing the risk of clinical development.

The pathophysiology of AD is known to be multi-factorial, and it is anticipated that combinations of DMTs with complementary or synergistic mechanisms of action may have an important therapeutic role, yet there is little opportunity for approval of a combination therapy by 2025. Increased focus on clinical development of combination therapies is likely a prerequisite for an optimal therapeutic benefit, as is evidenced by current state-of-the-art treatments for many cancers as well as HIV-AIDS, cardiovascular disease, and tuberculosis. The role of and challenges with combination therapies in AD have been recently highlighted by Hendrix et al. [16]. Challenges are many and include those related to dose finding, number of required studies (exacerbated in the study of combination therapies where traditionally a factorial design is required—combination is compared to the two monotherapy arms and to placebo) as well as strategic issues related to co-development of combinations of drugs that reside in different companies. Regulators are considering the impact of combination therapy; for example, they acknowledge that a full factorial design may be difficult for DMTs due to the large sample sizes required in each arm over long study periods and are open to considering alternatives, for example exclusion of the monotherapy arms, if scientifically justified [46]. This would reduce the required sample sizes thus hastening trial recruitment and clinical assessment.

#### Collaborations

Measures to speed up clinical trial execution and regulatory review will rely heavily on stakeholder collaborations and data sharing. There are numerous and increasing collaborations among relevant parties (pharma, government, academia, advocacy groups) and established collaborations are joining together.

In Europe, the Innovative Medicines Initiative (IMI) [54] aims to accelerate drug development by facilitating collaboration among key players involved in healthcare research, including universities, industry, patient organizations, and medicines regulators. IMI projects include: the Prediction of cognitive properties of new drug candidates for neurodegenerative diseases in early clinical development (PharmaCog) [55], to increase the ability to predict new medicines from laboratory studies and clinical models; the European Medical Information Framework Platform (EMIF) [56], to develop a framework for evaluating, enhancing and providing access to AD data (including CSF data, MRI scans, PET scans, plasma samples, DNA samples and RNA samples) from across Europe using Electronic Healthcare Record databases, and to identify biomarkers of AD onset in the preclinical and prodromal phase as well as for disease progression and to identify high-risk individuals for prevention trial participation; and the European Prevention of Alzheimer’s Dementia (EPAD) Initiative [57], to provide an environment for testing interventions targeted at delaying the onset of clinical symptoms with the aim of establishing a European-wide register of 24,000 participants.

The Critical Path Institute (C-Path) [58] is a non-profit organization that promotes collaboration across regulators, industry, and the research community. C-Path’s mission is to help catalyze the translation of scientific discoveries into innovative medicines, including in AD. Within C-Path is the Coalition Against Major Diseases (CAMD), which focuses on sharing precompetitive patient-level data from the control arms of legacy clinical trials, developing new tools, and developing consensus data standards. It has also led a process that successfully advanced a clinical trial simulation tool for AD through the formal regulatory review process at the FDA and EMA [59]. CAMD will integrate its AD clinical trial data into the Global Alzheimer’s Association Interactive Network (GAAIN) [60] to broaden GAAIN’s data sharing abilities. GAAIN is a platform for searching and integrating data from AD and other dementia research studies to assists scientists who are working to advance research and discovery in the field.

Accelerating Medicines Partnership-Alzheimer’s Disease (AMP-AD) [61] is an initiative between the National Institutes of Health (NIH), Pharma, and non-profit organizations. Its activities include the Biomarkers Project to explore the utility of tau imaging and novel fluid biomarkers for tracking responsiveness to treatment and/or disease progression, and the Target Discovery and Preclinical Validation Project aimed at shortening the AD drug development process through analysis of human tissue data and network modeling approaches.

The Alzheimer’s Prevention Initiative [62] is a collaboration focused on evaluating therapies in persons who are cognitively normal but at increased genetic risk of developing symptoms of AD. The Alzheimer’s Prevention Registry [17] is part of the initiative. The Generations study was launched through the Alzheimer’s Prevention Initiative and funded by NIH, Novartis, and other funding groups, and is designed to assess the ability of two investigational anti-amyloid therapies to prevent or delay the development of AD symptoms in a population known to be at high risk for the disease because of their age and apolipoprotein E epsilon 4 (ApoE4) gene status (APOE-e4 homozygotes).

The Dominantly Inherited Alzheimer Network Trials Unit (DIAN-TU) [18] is a collaboration between NIH, academic centers, industry, and the Alzheimer’s Association to advance tirals of new therapies in high risk populations and to help initiate meetings of key stakeholders, including patients, regulatory agencies, industry, and AD researchers and non-profit organizations. DIAN-TU also developed an expanded registry, as discussed above.

#### Big data

Modern biomedical data collection is generating exponentially greater amounts of information, and this can positively impact the timelines for development of new therapies for AD. The plethora of complex data poses significant opportunities to discover and understand the critical interplay among such diverse domains as genomics, proteomics, metabolomics, and phenomics, including imaging, biometrics, and clinical data [63]. Big data analytics are appropriate for interrogation of these data and the relationships among data to produce hypotheses regarding target identification, systems pharmacology, and drug development. Data sharing, causal inference and pathway-based analysis, crowdsourcing, and mechanism-based quantitative systems modeling represent successful real-world modeling opportunities where big data strategies can assist in identifying relationships, risk factors for onset and progression, and new outcome measures that could not be discovered with traditional analytic techniques [64]. Mechanism-based modeling, process and interaction simulation approaches, integrated domain knowledge, complexity science, and quantitative systems pharmacology can be combined with data-driven analytics to generate predictive actionable information for drug discovery programs, target validation, and optimization of clinical development [65]. These data are available now and are accumulating at great speed; application of appropriate bioinformatics analyses could produce discoveries capable of accelerating drug development timelines and assisting in meeting the 2025 goal. Big data will play a larger role in drug discovery and development in the post-2025 period.

#### Financing

AD drug development is hugely expensive. Global financing for AD research and development is inadequate relative to costs of the disease and the level of funding is low compared to other diseases with a significant public health impact. While countries are beginning to recognize the impact of AD costs of care on global economic growth and are making investments in this area, it is unrealistic to rely on public financing alone. To accelerate drug development efforts, more innovative approaches centered on a collaborative, cross-sector effort that links the search for a cure with the world’s private investment markets are needed. This will include: social impact investing so that private investors can pursue investment returns while supporting causes that reflect their values/priorities; venture capital and venture philanthropy, for example The Alzheimer’s Drug Discovery Foundation (ADDF) [66] and The Cure Alzheimer’s Fund [67]; crowd funding, for example Give To Cure [68] whose first campaign is targeting AD; state financing; industry investment in the pipeline of early stage companies; collaboration among drug companies for risk sharing; and academia-industry collaborations to support research for new target identification, early drug candidate identification, and creation of spin-off biotechnology companies. Investment in drug development could also be encouraged by legislative actions to further reduce risk and cost. For example, lengthy AD drug development shortens the time from approval to patent expiration, thus reducing the likelihood of recouping the expense of drug development, including failed efforts; additional patent life and tax incentives for developing treatments for chronic neurological disorders might make AD drug development more attractive to additional industry investors.

## Conclusion

Accelerating research and clinical development efforts and bringing DMTs to market sooner would have a significant impact on the future societal burden of AD. Under the current conditions, only drugs currently in late Phase 1 or later could be ready by 2025, and only if the studies progress optimally. If pipeline attrition rates remain high, it is likely that only a few compounds could possibly reach this milestone. There is a great need to reduce the time and risk of AD drug development to reach the 2025 goal.

We have discussed the key areas by which we can address this challenge—improvement in trial design; better trial infrastructure; disease registries of well-characterized patient cohorts to help with fast/timely enrollment of appropriate study population; validated biomarkers to better detect disease, determine risk and monitor disease as well as predict disease response; more sensitive clinical assessment tools; and faster regulatory review.

To implement change requires efforts to build awareness, educate and foster engagement; financial commitment to increase funding for both basic and clinical research; collaboration to reduce fragmented environments and systems, to increase learnings from successes and failures, to promote data standardization and thus increase wider data sharing; and a greater depth of understanding of AD at the basic biology level and speedy translation of new knowledge into clinical development. Improved mechanistic understanding of disease development and progression is critical to more efficient AD drug development and will lead to improved therapeutic approaches and targets. More effective tools, such as biomarkers and sensitive cognitive assessments, and more appropriate selection of participants will lead to improved clinical trials. The effort required to advance a drug from bench to bedside is poorly understood by most AD stakeholders, and education regarding the complexities, long time frames, and expense of AD drug development is critical. As these steps are put in place and plans come to fruition (e.g., approval of a DMT), it can be predicted that momentum will build, the process will be self-sustaining, and the path to 2025, and beyond, will become clearer.

## Abbreviations

A4: Anti-Amyloid Treatment in Asymptomatic AD
AD: Alzheimer’s disease
ADAS-Cog: Alzheimer’s Disease Assessment Scale-Cognitive subscale
ADCOM: AD composite score
ADCS: Alzheimer’s Disease Cooperative Study
ADDF: Alzheimer’s Drug Discovery Foundation
ADL: Activities of daily living
AMP-AD: Accelerating Alzheimer’s Research and Drug Development-Alzheimer’s Disease
ApoE4: Apolipoprotein E epsilon 4
BADL: Basic activities of daily living
CAMD: Coalition Against Major Diseases
CDR-SB: Clinical Dementia Rating-Sum of Boxes
C-Path: Critical Path Institute
CSF: Cerebrospinal fluid
DFC: Dementia Friendly Community
DIAN-TU: Dominantly Inherited Alzheimer Network Trials Unit
DMT: Disease-modifying therapy
EMA: European Medicines Agency
EMIF: European Medical Information Framework Platform
EPAD: European Prevention of Alzheimer’s Dementia
FCSRT: Free and Cued Selective Recall Reminding Test
FDA: Food and Drug Administration
GAAIN: Global Alzheimer’s Association Interactive Network
GAP: Global Alzheimer’s Platform
HCP: Healthcare professional
IADL: Instrumental activities of daily living
iADRS: Integrated Alzheimer’s Disease Rating Scale
IMI: Innovative Medicines Initiative
iPS: Induced pluripotent stem
IRB/EC: Institutional review board/ Ethics Committee
IWG: International Working Group
MCI: Mild cognitive impairment
MMSE: Mini-Mental State Examination
MRI: Magnetic resonance imaging
NAPA: National Alzheimer’s Project Act
NIA-AA: National Institute on Aging-Alzheimer’s Association
NIH: National Institutes of Health
PACC: Preclinical Alzheimer Cognitive Composite
PDUFA: Prescription Drug User Fee Act
PET: Positron emission tomography
USC ATRI: University of Southern California Alzheimer’s Therapeutic Research Institute
